# Which Matters More: Intention or Outcome? The Asymmetry of Moral Blame and Moral Praise

**DOI:** 10.3390/bs15091265

**Published:** 2025-09-16

**Authors:** Zhi-Meng Li, Lin Xiao, Hong-Yue Sun

**Affiliations:** School of Psychology, Shanghai Normal University (SHNU), Shanghai 200234, China; 1000533108@smail.shnu.edu.cn (Z.-M.L.); liberfree@163.com (L.X.)

**Keywords:** behavioural intention, behavioural outcome, moral blame, moral praise, dual-process theory

## Abstract

This study investigated the asymmetrical effects of intentions and outcomes on moral blame and praise within scenarios involving harm and help. By manipulating self–other perspective differences and the severity of outcomes, it further explored their moderating roles in these asymmetrical effects. The key findings include the following: (1) Intention and outcome asymmetrically influenced moral blame and praise: moral blame prioritized intentions, whereas moral praise emphasized outcomes. (2) Self–other perspectives moderated the asymmetric effects of behavioural intentions and outcomes on moral blame but did not moderate the asymmetric effect on moral praise: from the perspective of others, blame tended to focus on intentions, while the self-perspective prioritized outcomes. (3) Outcome severity moderated the effect of behavioural intentions on moral blame and moral praise. Compared to severe outcomes, intention was a stronger predictor of blame and praise when the outcome was minor; however, this moderating effect was specifically observed for moral blame from others’ perspectives and for moral praise from self-perspective.

## 1. Introduction

Moral judgement refers to the process by which individuals apply moral concepts and knowledge to evaluate the rightness, wrongness, or ethical standing of a certain behaviour or subject (Song et al., 1989). People often make moral judgements about various social events, praising prosocial behaviours such as donations and condemning behaviours such as breach of trust and environmental destruction. However, when behavioural intentions and outcomes are inconsistent (e.g., unintentional harm or accidental benefits), individuals exhibit distinct moral evaluations. Various models treat intentions and outcomes as key factors in making moral judgements (Duan et al., 2012; Gan et al., 2018; Cushman et al., 2013; Young et al., 2007), including the Culpable Control Model (Alicke, 2000), Path Model of Blame (Malle et al., 2014), and Dual Process Model (Cushman, 2008), all of which integrate intentions and outcomes into the process of moral blame. Currently, much of the research on the impacts of behavioural intentions and outcomes on moral judgements has concentrated on the area of moral blame. The research explores the prioritized role of intentions and contrasts the different effects of these two factors (Cushman, 2008; Cushman, 2015; Young et al., 2011). Although the asymmetry between moral blame and praise in multiple aspects has been well documented (Bostyn & Roets, 2016; Cushman & Greene, 2012; Pizarro et al., 2003), no studies have yet compared intentions and outcomes in terms of the relative moral importance of blame and moral praise, i.e., if moral blame values intentions more, does moral praise also value intentions more, or does it value outcomes more? Do the bases for our moral judgements change when we experience moral events firsthand, when we are bystanders, and when the outcome severity ranges from minor benefits to life-threatening situations? Therefore, this study attempts to compare the effects of intentions and outcomes in moral praise and moral blame to expand the research on the asymmetry between moral blame and moral praise and its moderating mechanisms.

### 1.1. Intentions and Consequences in Moral Judgement

In the field of moral judgement, although individuals make moral evaluations based on diverse criteria, they consider mainly the actor’s intentions and the consequences of that action (Greene & Haidt, 2002). Intent refers to the actor’s mental state when choosing and planning an act, which involves a comprehensive understanding of the action’s goals and consequences (Malle & Knobe, 1997). The consequences of the action refer to the perceived severity of the harm inflicted by the actor (Cushman, 2008). Among moral events, intentions and outcomes do not always coincide.

### 1.2. Asymmetric Effects of Intentions and Outcomes on Moral Judgements

As research on moral blame and praise intensifies, it becomes increasingly evident that praise and blame are not mirror images of each other, nor are they the products of a unified cognitive process. Negative events and stimuli evoke stronger psychological reactions and more in-depth thinking than positive events do (Baumeister et al., 2001; Rozin & Royzman, 2001). Such “negative bias” has been found in the areas of loss aversion (Kahneman & Tversky, 1984), impression formation (Peeters & Czapinsky, 1990), and moral judgement (Bostyn & Roets, 2016). For example, research has demonstrated that people have different criteria for morally evaluating negative and positive behaviours. Individuals are significantly sensitive to criteria such as behavioural intent and controllability when determining whether to assign blame; however, these factors seem to play a less significant role in the mechanisms of praise (Ohtsubo, 2007; Pizarro et al., 2003).

Error management theory (Green & Swets, 1996) proposes that the costs related to incorrect moral blame and praise are asymmetric in moral judgement processes. From a social-functioning perspective, the effects of intentions on negative behaviours are more prominent (Gan et al., 2018). When behaviours result in negative outcomes, actors who intentionally violate moral norms receive harsher moral blame than those who do not (Moran et al., 2011; Young et al., 2007). Compared with blame, praise is relatively cost-free. People generally enjoy being praised, and the consequences of wrongly praising an actor are relatively minor for both the praiser and the recipient. That is, whether the praise is intention-based or outcome-based, it can build a good relationship between them. Anderson et al. (2020) and Pizarro et al. (2003) demonstrated asymmetry in the reinforcing effects of intentions in moral praise and blame. They reported that positive behaviours do not receive the same careful consideration as negative behaviours do, and there is no significant difference between individuals’ praise for intentional and unintentional positive behaviours. In other words, blame often needs to be justified. It is crucial to adjust blame judgements according to the actor’s causality, intent, and magnitude of the behaviour’s outcome, while praise does not adjust judgement (Malle et al., 2022). Accordingly, we argue that there is asymmetry in the roles of intention and outcome in moral blame and moral praise and propose the following hypotheses:

**Hypothesis 1.**  *People focus more on behavioural intentions in the process of moral blame than on behavioural outcomes.*

**Hypothesis 2.**  *People focus more on behavioural outcomes during moral praise than on behavioural intentions.*

### 1.3. Self–Other Perspective and Moral Judgement

Differences in self–other perspectives have been widely explored in decision-making research (Liu et al., 2010; Sun et al., 2016; Polman, 2012; Trope & Liberman, 2000). Similarly, self–other differences are evident in moral judgement, particularly in moral dilemma situations (Agerström et al., 2013; Trope & Liberman, 2000; Sun et al., 2016). Specifically, individuals taking an other-focused perspective tend to be more utilitarian, whereas those taking a self-focused perspective tend to be more moralistic. Current research on moral praise and blame is conducted mainly from the observer’s perspective rather than the first-person perspective. However, in real-world contexts, an individual’s criteria for making blame judgements may differ when oneself is the victim versus when another person is the victim.

Construal level theory (Trope & Liberman, 2000) posits that individuals employ different levels of abstract representation when making decisions for themselves than when making decisions for others. When making decisions for others, individuals think at a higher level of abstraction, typically focusing more on the event’s completeness and its core features and inclining towards a rule-based orientation. In contrast, when making decisions for themselves, individuals operate at a lower level of abstraction, concentrating more on details and favouring an outcome-based orientation. According to construal level theory, in an ethical violation situation, the third-party perspective focuses more on the causes leading to the violation, whereas the victim’s perspective focuses more on the consequences of the violation (Lammers, 2012).

As previously noted, people may focus more on behavioural outcomes in the process of moral praise. Isen and Means (1983) found that positive affect prompts individuals to adopt an “outcome heuristic” decision-making strategy. When adopting a first-person perspective, specifically when individuals personally experience a positive outcome (e.g., “He helped me solve the problem”), positive affect enhances this outcome-prioritizing strategy. Individuals may even actively disregard ambiguous intentions (e.g., “He might have just helped coincidentally”). This cognitive bias similarly applies to moral praise evaluations driven by positive affect. Consequently, self–other perspective differences may not be significant in the context of moral praise.

In summary, we hypothesize that the self–other perspective plays a moderating role in the effect of behavioural intentions and outcomes on moral blame, but not on moral praise. Accordingly, we propose the following:

**Hypothesis 3.**  *In moral blame, intentions more strongly predict blame under others’ perspectives than under self-perspective; Outcomes more strongly predict blame under self-perspective than under others’ perspectives.*

**Hypothesis 4.**  *In moral praise, outcome is a stronger predictor than intention across both self- and others’ perspectives.*

### 1.4. Severity of Harm and Moral Judgement

The severity of harm is a critical variable influencing moral judgements. Numerous studies have emphasized the relationship between harm severity and moral judgements. In general, in moral scenarios, as the severity of harm suffered by the victim increases, observers tend to make harsher judgements (Schein & Gray, 2018; Schwartz et al., 2022; Lowe & Medway, 1976). The impact of outcome severity on moral judgements also extends to research on moral dilemmas, in which participants make judgements about whether it is acceptable to sacrifice one person to save many, and studies have shown that the closer the number of deaths is to the number of people saved, that is, the smaller the ratio of the two, the less acceptable such a sacrifice becomes (Costa-Lopes et al., 2021; Mata, 2019). This implies that outcome severity dominates moral judgements in high-stakes scenarios (e.g., life-and-death decisions).

The increasing severity of judgements as negative outcomes worsen is consistent with a justice system where individuals should be punished in proportion to the harm they have inflicted. When making liability judgements, judges employ differentiated criteria for cases with significantly different outcome severity levels, such as minor property damage versus personal injury resulting in disability. More forgiveness is given for unintentional behaviours that result in mildly injurious outcomes than for those resulting in severe outcomes. The affective overload associated with severe injuries may limit intention-based analysis. That is, individuals have a “threshold of injury severity.” Above this threshold, the contribution of behavioural intent to judgements of unintentional moral violations is reduced (Schwartz et al., 2022).

Concurrently in the domain of moral praise, Wang et al. (2024) found that even if helpers publicly showcase donations on social media (a behaviour potentially perceived as showing off), observers still praise altruistic acts primarily based on outcomes of the actual donation amount. For instance, donations of CNY 1000 received significantly higher praise than donations of CNY 100, regardless of perceived exhibitionist intent. This indicates that observers prioritize the tangible value of benevolent acts over the purity of underlying motives.

Accordingly, we hypothesize that outcome severity moderates the effects of behavioural intentions and outcomes on both moral blame and praise and propose Hypothesis 5: In judgements of both moral blame and moral praise, as outcome severity increases, the tendency to rely on intentions in judgements weakens, while the tendency to rely on outcomes strengthens.

This paper tested these hypotheses through three studies. Study 1 explored the effects of intentions and outcomes on moral evaluations in harming versus helping situations and explored the asymmetry of the effects of intentions and outcomes on moral blame and moral praise. Studies 2 and 3 adapted story materials from Study 1 to explore the moderating effects of intentions and outcomes on moral blame and moral praise by manipulating the differences in participants’ perspectives and the extent of outcomes.

## 2. Study 1: Asymmetric Effects of Behavioural Intentions and Outcomes on Moral Blame and Praise

### 2.1. Experiment 1a: Effects of Behavioural Intentions and Outcomes on Moral Blame in Hurtful Situations

Experiment 1a: A self-administered moral blame judgement questionnaire was developed by adapting materials from previous studies on harmful situations to compare the effects of behavioural intention and outcome on moral blame in these contexts.

#### 2.1.1. Pre-Experiment 1a: Screening of Moral Blame Materials

Eight story scenarios were selected from the moral blame situations used in previous research (Young et al., 2007), namely, bicycle, ham sausage, protective gear, pond, insecticide, curling iron, sushi, and sneakers. To make the intention and outcome scores more discriminative, four groups were set up in the formal experiment: (1) no harm (neutral intention, neutral outcome), (2) accidental harm (neutral intention, negative outcome), (3) attempted harm (negative intention, neutral outcome), and (4) effective harm (negative intention, negative outcome). Therefore, in Pre-experiment 1a, eight story scenarios were screened via a web-distributed questionnaire, with each participant randomly assigned to one of four groups. A total of 217 samples were collected (76 males; 141 females; range: 21.59 ± 4.10 years). All participants received compensation for completing the questionnaires and passing the main examiner’s review.

**Figure 1:**
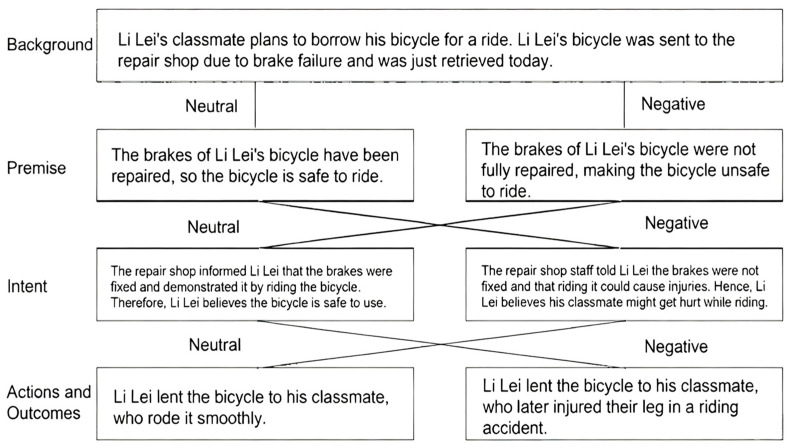
Moral blame scenario (taking the “bicycle” scenario as an example).

The Moral Blame Judgement Questionnaire consists of five sections, as follows:

(1) Demographic Information.
(2) Story Scenarios: To standardize the story materials, each story scenario was presented in segments following the order of background-foreshadowing-behavioural intention-behavioural outcome. Each story scenario had four conditions, and an example of the experimental material (using the bicycle material as an example) is shown in Figure 1.
(3) Comprehension Questions. Two comprehension questions were designed in this experiment to confirm whether participants could correctly infer the intentions of the story’s protagonist and the outcome experienced by another person in the story from each story scenario (Kurdi et al., 2020). Intentional comprehension questions include whether the protagonist believes that something bad will happen. The outcome comprehension question asks whether something bad happens to the other person in the story.
(4) Intentionality Question. The story protagonist’s intentions were tested for manipulativeness on a 7-point scale (1 = completely unintentional, 7 = completely intentional), where larger numbers represent more negative intentions (Martin & Cushman, 2016).
(5) Outcome-based Questions. A manipulative test of the outcome experienced by the other person in the story was carried out on a 7-point scale (1 = no harm, 7 = maximum harm), where a larger number represents a more negative outcome (Martin & Cushman, 2016).

A one-sample *t* test of the mean scores of the intentional questions and the mean scores of the outcome questions against the median of the scores (4) indicated that the negative intent scores (i.e., high intent scores) were not significantly different from the median in the protective-gear and pond story scenarios (*p* = 0.067; *p* = 0.493). This suggested that the manipulation of negative intent failed in these scenarios, so the protective gear and pond scenes were excluded. In Experiment 1a, six story situations were used: bicycle, ham sausage, insecticide, curling iron, sushi, and sneakers.

#### 2.1.2. Participants

In this experiment, the questionnaire was distributed to participants through the “Wenjuanxing” platform. After screening participants (excluding minors and repeated respondents), a total of 623 valid samples were collected (269 males, 354 females; age range: 21.92 ± 4.22 years). The questionnaires were distributed online via encrypted links. Participants first read the “Informed Consent Form” after clicking the link and entered the experiment after confirming their consent. The experiment adopted a “scenario-by-scenario presentation” mode, where each story scenario was displayed on an independent page. Participants had to correctly complete the comprehension questions of the previous scenario to proceed to the next one, so as to avoid data deviation caused by inattention. All participants were paid after completing the questionnaire and passing the review of the questionnaire by the main examiner.

Like in the pre-experiment, the participants were randomly assigned to one of four experimental conditions: (1) no harm (neutral intention, neutral outcome), (2) accidental harm (neutral intention, negative outcome), (3) attempted harm (negative intention, neutral outcome), and (4) effective harm (negative intention, negative outcome). Neutral and negative intentions and outcomes were set up to enhance the discriminability of the intention and outcome scores. The intention ratings and outcome ratings were used as predictor variables for moral blame. Each participant read six story materials: bicycle, ham sausage, insecticide, curling iron, sushi, and sneakers. After reading each story, the participants were required to complete comprehension, intention, and outcome questions related to the corresponding material and to rate the moral blameworthiness of the protagonist’s behaviour.

#### 2.1.3. Results

The relationships among intentions, outcomes, and moral blame ratings across story scenarios were examined. To determine whether intentions and outcomes significantly influence moral blame evaluations in story scenarios, a linear regression analysis was conducted with intentions and outcomes as predictors of moral blame. A standard regression analysis was performed with moral blame scores as the dependent variable and intention and outcome ratings as the independent variables. The specific results of the regression model are shown in Table 1. Intention significantly predicted moral blame scores across scenarios (*p*s < 0.001), with more negative intentions leading to stronger blame; the positive predictive effect of the outcome on moral blame scores was significant (*p*s < 0.001), meaning that the more negative the outcome was, the greater the degree of moral blame.

Relationships among intention, outcome, and moral blame ratings: A standardized regression was conducted, with the mean score of moral blame across the six scenarios as the dependent variable and intention and outcome ratings as the independent variables. Intention significantly predicted moral blame (*β* = 0.70, 95% CI [0.68, 0.72], *t* (620) = 87.91, *p* < 0.001), and the more negative the intention was, the greater the degree of moral blame received. Outcome was a significant positive predictor (*β* = 0.26, 95% CI [0.24, 0.28], *t* (620) = 31.82, *p* < 0.001), and the more negative the outcome was, the greater the degree of moral blame received. According to previous studies, nonoverlapping confidence intervals for standardized regression coefficients indicate a significant difference (Cumming, 2009). Since the confidence intervals of the regression coefficients of intention and those of the outcome variable were nonoverlapping, intention was a stronger predictor than the outcome in the evaluation of moral blameworthiness.

By setting α = 0.05 with a sample size of 623 and calculating the effect size in each scenario, post hoc tests revealed that the achieved statistical power reached 1 across all scenarios.

By restricting the model to equate the path coefficients of intention and outcome in moral blame and comparing the two models ($\chi^{2}$ = 1159.54, DIF = 0.46, *p* < 0.001), the null hypothesis was rejected. This finding indicates that the paths of intention and outcome were significantly different, regardless of whether the perspective was from others or the self.

### 2.2. Experiment 1b: The Effect of Behavioural Intentions and Outcomes on Moral Praise in Helping Situations

This experiment investigated the joint influence of behavioural intentions and outcomes on moral praise in helping situations. It adapted materials from previous studies and utilized a self-administered Moral Praise Judgement Questionnaire.

#### 2.2.1. Pre-Experiment 1b: Screening of Moral Praise Materials

The pre-experiment employed six moral praise story situations (Gan et al., 2016), namely, necklace, restaurant, swimming pool, ski resort, amusement park, and hospital. Questionnaires were distributed to the participants via the internet. A total of 242 samples were collected (96 males; 146 females; age range: 22.85 ± 4.56 years), and the experimental procedure was identical to that of pre-experiment 1a. The Moral Praise Judgement Questionnaire used in this experiment had five sections. In section (5)—Outcome Questions, a manipulative test of the outcome experienced by the other person in the story was conducted on a 7-point scale (1 = No help, 7 = Maximum help).

The results showed that participants were better able to infer the corresponding intention and outcome information based on the moral praise story scenarios; thus, a total of six story scenarios, namely, necklace, restaurant, swimming pool, ski resort, amusement park, and hospital, were used in Experiment 1b.

#### 2.2.2. Participants

In this experiment, the questionnaire was distributed to participants via the internet. A total of 628 samples (239 males; 389 females; age range: 21.72 ± 3.84 years) were collected, and all the participants were paid after completing the questionnaire and passing the review of the questionnaire by the main examiner. The recruitment and collection procedures for participants were the same as those in Experiment 1a.

#### 2.2.3. Experimental Design and Procedures

Six helping scenarios were employed: necklace, restaurant, swimming pool, ski resort, amusement park, and hospital. The experimental design and procedures were identical to those in Experiment 1a. The Moral Praise Judgement Questionnaire used in this experiment had six sections: (1) demographic information, (2) story situations, (3) comprehension questions, (4) intention questions, and (5) outcome questions, which were the same as those used in Pre-experiment 1b. With respect to (6) moral praise evaluation, taking the “necklace” scenario as an example, the moral praise evaluation question was as follows: To what extent should Liang Ke’s behaviour be praised? The participants were required to make a choice on a 7-point scale (1 = no praise, 7 = maximum praise).

#### 2.2.4. Results

The relationships among intention, outcome, and moral praise ratings across different story scenarios were examined. To determine whether intention and outcome significantly predicted moral praise in the six scenarios, a linear regression analysis was conducted, with intention and outcome serving as predictors of moral praise. A standard regression analysis was performed, with moral praise scores as the dependent variable and intention and outcome ratings as the independent variables. The specific results of the regression model are shown in Table 1. Intention significantly predicted moral praise scores (*p*s < 0.001). That is, the more positive the intention was, the greater the degree of moral praise. The positive predictive effect of outcome on moral praise scores was also significant (*p*s < 0.001), indicating that the more positive the outcome was, the greater the degree of moral praise.

Relationships among intention, outcome, and moral praise ratings: A standardized regression was conducted, with the mean moral praise score across the six scenarios as the dependent variable and the intention and outcome ratings as independent variables. The results revealed that intention was a significant predictor of moral praise (*β* = 0.44, 95% CI [0.43, 0.46], *t* (628) = 59.78, *p* < 0.001). That is, more positive intentions were associated with higher moral praise ratings. Similarly, outcome was a significant predictor (*β* = 0.51, 95% CI [0.50, 0.53], *t* (628) = 67.32, *p* < 0.001), meaning that more positive outcomes were associated with higher moral praise scores.

By setting α = 0.05 with a sample size of 628 and calculating the effect size in each scenario, post hoc tests revealed that the achieved statistical power reached 1 across all scenarios.

By restricting the model to equate the path coefficients of intention and outcome in moral praise and comparing the two models ($\chi^{2}$ = 1276.48, DIF = −0.09, *p* < 0.001), the null hypothesis was rejected. This finding indicates that there was a significant difference between the paths of intention and outcome.

**Table 1:** Linear regression models predicting moral blame/praise from intention and outcome ratings across six scenarios.

Types of Evaluation	Situation	Predictors	*β*	95% CI	*t*	*R* ^2^	*F*
Moral Blame (Experiment 1a)	Bicycle					0.71	754.42 ***
		Intention	0.71	[0.67, 0.75]	34.38 ***		
		Outcome	0.23	[0.18, 0.27]	10.69 ***		
	Ham Sausage					0.72	811.13 ***
		Intention	0.66	[0.62, 0.70]	32.64 ***		
		Outcome	0.28	[0.24, 0.32]	12.87 ***		
	Insecticide					0.75	943.61 ***
		Intention	0.69	[0.65, 0.73]	35.95 ***		
		Outcome	0.29	[0.25, 0.32]	15.04 ***		
	Hair Curling Iron					0.76	999.62 ***
		Intention	0.69	[0.65, 0.73]	34.94 ***		
		Outcome	0.28	[0.24, 0.31]	13.90 ***		
	Sashimi					0.79	1148.98 ***
		Intention	0.74	[0.70, 0.77]	40.58 ***		
		Outcome	0.23	[0.20, 0.27]	12.53 ***		
	Sneakers					0.77	1043.01 ***
		Intention	0.70	[0.66, 0.74]	36.62 ***		
		Outcome	0.25	[0.21, 0.29]	12.88 ***		
	Six Scenarios’ Average					0.75	5656.89 ***
		Intention	0.70	[0.68, 0.72]	87.91 ***		
		Outcome	0.26	[0.24, 0.28]	31.82 ***		
Moral Praise (Experiment 1b)	Necklace					0.71	765.23 ***
		Intention	0.48	[0.44, 0.52]	24.84 ***		
		Outcome	0.48	[0.44, 0.51]	24.54 ***		
	Restaurant					0.76	965.37 ***
		Intention	0.45	[0.42, 0.49]	24.43 ***		
		Outcome	0.51	[0.47, 0.55]	27.85 ***		
	Swimming Pool					0.73	855.50 ***
		Intention	0.45	[0.41, 0.49]	24.65 ***		
		Outcome	0.52	[0.48, 0.55]	27.59 ***		
	Ski Resort					0.75	959.72 ***
		Intention	0.42	[0.39, 0.46]	24.62 ***		
		Outcome	0.52	[0.49, 0.56]	28.52 ***		
	Amusement Park					0.75	935.80 ***
		Intention	0.45	[0.42, 0.48]	25.88 ***		
		Outcome	0.51	[0.47, 0.54]	27.58 ***		
	Hospitals					0.76	986.56 ***
		Intention	0.39	[0.35, 0.42]	22.07 ***		
		Outcome	0.55	[0.51, 0.59]	29.50 ***		
	Six Scenarios’ Average					0.74	5415.42 ***
		Intention	0.44	[0.43, 0.46]	59.78 ***		
		Outcome	0.51	[0.50, 0.53]	67.32 ***		

All the data were standardized. *** *p* < 0.001.

The results of Study 1 indicate that people place more importance on the intention factor when making moral blame evaluations, whereas moral praise evaluations rely more on the outcome factor. The findings of this study support Hypotheses 1 and 2. The results of Study 1 demonstrate that there is an asymmetrical effect of behavioural intentions and outcomes on moral blame and praise.

However, Study 1 only measured blame and praise evaluations from others’ perspectives; therefore, Study 2 further explored whether perspective differences moderate the relationships between intentions and outcomes and between blame and praise.

## 3. Study 2: The Effects of Intentions and Outcomes on Moral Blame and Praise: The Moderating Role of Self–Others

Experiment 2 builds on Experiment 1 by further exploring whether there are differences in the effects of self–other perspectives of behavioural intentions and outcomes on moral blame and moral praise by varying the narrator’s persona perspective in wounding story situations.

### 3.1. Experiment 2a: The Effects of Intentions and Outcomes on Moral Blame: The Moderating Role of Self–Others

#### 3.1.1. Participants

In this experiment, the questionnaire was distributed to the participants via the internet. A total of 592 samples were collected: 296 in the “others” group and 296 in the “self” group (244 males; 348 females; age range: 21.98 ± 3.70 years), and all participants were compensated for completing the questionnaires and for passing the examination of the questionnaires by the main examiner. The recruitment and collection procedures for participants were the same as those in Experiment 1a.

#### 3.1.2. Experimental Design and Procedures

Experiment 2a adopted a 2 (Intention: Neutral vs. Negative) × 2 (Outcome: Neutral vs. Negative) × 2 (Perspective: Self vs. Other) between-subjects design. This experiment employed two harmful scenarios from Experiment 1a, namely, pesticides and sushi as materials. Neutral and negative intentions and neutral and negative outcomes were set up to make intention ratings and outcome ratings more discriminatory. The intention ratings and outcome ratings were used as predictor variables of moral blame in subsequent analyses. After reading each item, the participants completed comprehension, intention, and outcome questions related to the corresponding material and evaluated the moral blameworthiness of the main character’s behaviour.

Taking the “insecticide” scenario as an example. Neutral intention: Sun Yao believed that the neighbour was not at home (e.g., the neighbour informed her that they would go out early on Saturday) and knew that spraying insecticide would not cause harm; Negative intention: Sun Yao believed that the neighbour was at home (e.g., knowing that the neighbour rarely goes out on weekends), and knew that spraying insecticide would cause harm.

Neutral outcome: The neighbour was not affected at all; Negative outcome: The neighbour suffered from acute toxic shock due to inhaling the insecticide.

Other’s perspectives: Evaluate from a third-party perspective: “To what extent should Sun Yao be blamed for her behaviour?”; Self-perspective: “If you were Sun Yao’s neighbour, would you blame Sun Yao for her behaviour?”.

#### 3.1.3. Results

Relationships among intention, outcome, and blame ratings from the self–other perspective: Hierarchical regression analysis was used to examine the moderating effect of perspective (self vs. other) on the influence of intention and outcome on moral blame, with standardized blame ratings as the dependent variable. The specific results of the regression model are shown in Table 2. The overall model test revealed that adding interaction terms significantly improved model fit (Δ*R*^2^ = 0.023, *F*(3,585) = 21.86, *p* < 0.001).

Analysis of interaction effects revealed that the Intention × Perspective interaction term was significant (*p* < 0.001). Simple slope tests further indicated that intentions more strongly predicted blame in the others’ perspectives condition compared to the self-perspective condition (*p*s < 0.001); The Outcome × Perspective interaction term was significant (*p* < 0.001). Simple slope tests further showed that outcomes more strongly predicted blame in the self-perspective condition compared to the others’ perspectives condition (*p*s < 0.001). This indicated that individuals focus more on outcomes when evaluating their own behaviours but prioritize intentions when evaluating others’ behaviours.

Subgroup analyses were performed using a standardized regression. The self–other perspective was considered, with intention scores and outcome scores as independent variables and moral blame scores as the dependent variable.

Intention was a significant positive predictor of moral blame in the “others” group ($\beta_{insecticide}$ = 0.58, $\beta_{sushi}$ = 0.65, *p*s < 0.001), and outcome was also a significant positive predictor of moral blame ($\beta_{insecticide}$ = 0.36, $\beta_{sushi}$ = 0.33, *p*s < 0.001). Similarly, in the “self” group, intention was a significant positive predictor of moral blame ($\beta_{insecticide}$ = 0.40, $\beta_{sushi}$ = 0.41, *p*s < 0.001), and outcome was a significant positive predictor of moral blame ($\beta_{insecticide}$ = 0.51, $\beta_{sushi}$ = 0.54, *p*s < 0.001).

A standardized regression was performed with the mean moral blame score across both scenarios as the dependent variable. In regressions where moral blame was the dependent variable, the positive predictive effect of intention was significant from both the others’ perspective and the self-perspective. The more negative the intention was, the higher the blame score. The positive predictive effect of the outcome was also significant, and the more negative the outcome was, the higher the blame score. Moreover, the 95% confidence intervals for the intention and outcome coefficients were nonoverlapping under either perspective. From the others’ perspective, intention was a stronger predictor than outcome, whereas from the self-perspective, outcome was a stronger predictor than intention.

Post hoc power analysis (α = 0.05, sample size = 296) indicated that achieved statistical power reached 1 for both the Others-Blame and Self-Blame conditions.

By restricting the model to equate the path coefficients of intention and outcome in moral blame and comparing the two models, the null hypothesis was rejected for both the others’ perspective ($\chi^{2}$ = 714.92, DIF = −0.12, *p* < 0.001) and the self-perspective ($\chi^{2}$ = 787.33, DIF = 0.19, *p* < 0.001), which indicates that the paths of intention and outcome were significantly different from each other.

The results of Experiment 2a revealed that the coefficient of the explanatory effect of behavioural intention on moral blame evaluation was significantly greater when the stories were narrated in the third person, i.e., the other person’s perspective, and that people were more morally blameful on the basis of intention; moreover, the coefficient of the explanatory effect of behavioural intention on moral blame evaluation was significantly greater when the stories were narrated in the first person, i.e., the self’s perspective, and that people were more morally blameful on the basis of the outcome. Therefore, the self–other perspective moderates the effect of behavioural intentions—the outcome of moral blame, supporting Hypothesis 3.

### 3.2. Experiment 2b Effects of Intentions and Outcomes on Moral Praise: The Moderating Role of Self–Others

#### 3.2.1. Participants

In this experiment, the questionnaire was distributed to the participants via the internet. A total of 543 samples were collected, with 277 in the “others” group and 266 in the “self” group (176 males; 367 females; age range: 22.10 ± 5.35 years), and all the participants were paid accordingly after completing the questionnaire and passing the review of the questionnaire by the main examiner. The recruitment and collection procedures for participants were the same as those in Experiment 1a.

#### 3.2.2. Experimental Design and Procedures

Experiment 2b utilized the same two helping situations as did Experiment 1b (restaurant and ski resort). The experimental design and procedures were identical to those in Experiment 2a.

Taking the “ski resort” scenario as an example. Neutral intention: Zhou Yan believes that the young man is a professional skier who can easily bypass the garbage without any danger; Positive intention: Zhou Yan believes that the young man is a beginner skier who cannot bypass the garbage and is in danger.

Neutral outcome: The young man encounters no danger (e.g., he is a professional skier himself and does not need help); Positive outcome: The young man avoids the garbage due to Zhou Yan’s reminder, thus preventing a fall injury.

Others’ perspectives: Evaluate from a third-party perspective: “To what extent should Zhou Yan’s behaviour be praised?”; Self-perspective: “If you were the young person, would you praise Zhou Yan’s behaviour?”

#### 3.2.3. Results

Hierarchical regression analysis was used to examine the moderating effect of perspective (self vs. other) on the influence of intention and outcome on moral praise, with standardized praise ratings as the dependent variable. The specific results of the regression model are shown in Table 2. The overall model test revealed that adding interaction terms significantly improved the model fit (Δ*R*^2^ = 0.001, Δ*F*(3,536) = 5.30, *p* = 0.001), but small in effect size.

Main effects showed outcomes more strongly predicted praise than intentions (*p*s < 0.001). The Intention × Perspective interaction term was not significant (*p* = 0.69), and the Outcome × Perspective interaction term was not significant (*p* = 0.23). This indicated that the self–other perspective did not significantly moderate the effects of intention or outcome on moral praise.

Subgroup analyses were performed using a standardized regression. The self–other perspective was considered, with intention scores and outcome scores as independent variables and moral praise scores as the dependent variable.

Intention was a significant positive predictor of moral praise in the “others” group ($\beta_{restaurants}$ = 0.35, $\beta_{ski\;resorts}$ = 0.36, *p*s < 0.001), and the outcome was a significant positive predictor of moral praise ($\beta_{restaurants}$ = 0.52, $\beta_{ski\;resorts}$ = 0.58, *p*s < 0.001). In the “self” group, intention was a significant positive predictor of moral praise ($\beta_{restaurants}$ = 0.37, $\beta_{ski\;resorts}$ = 0.35, *p*s < 0.001), and outcome was a significant positive predictor of moral praise ($\beta_{restaurants}$ = 0.56, $\beta_{ski\;resorts}$ = 0.60, *p*s < 0.001).

A standardized regression was performed with the mean moral praise scores in both contexts as the dependent variable. In regressions with moral praise as the dependent variable, the positive predictor of intention was significant from both the others’ perspective and the self-perspective, and the more positive the intention, the higher the praise score. The positive predictor of outcome was significant, and the more positive the outcome, the higher the praise score. Outcome was a stronger predictor than intention in both the others’ perspective and the self-perspective, with no overlap in confidence intervals. In addition, the predictive coefficient of the outcome factor was greater for praise ratings from the self-perspective than from the others’ perspective.

By setting α = 0.05, employing sample sizes of 277 (Others-Blame) and 266 (Self-Blame), and calculating condition-specific effect sizes, post hoc tests revealed that statistical power reached 1 in both conditions.

By restricting the path coefficients of intention and outcome on moral praise to be equal through the restriction model, a comparison of the two models revealed that for the others’ perspective ($\chi^{2}$ = 680.29, DIF = −0.19, *p* < 0.001) and for the self’s perspective ($\chi^{2}$ = 754.80, DIF = −0.22, *p* < 0.001) and that for both the others’ and self’s perspectives in the evaluation of moral praise, the intentions and outcomes were not equal in their predictive power for moral praise, with the outcome being a stronger predictor than the intention.

**Table 2:** Linear regression model of intention and outcome scores for self–other moral blame/praise ratings.

	Types of Evaluation	Predictors	*β*	95% CI	*t*	*R* ^2^	*F*
Moral Blame (Experiment 1a)	Others-Blame					0.77	997.81 ***
		Intention	0.62	[0.58, 0.65]	32.41 ***		
		Outcome	0.35	[0.31, 0.38]	17.57 ***		
	Self-Blame					0.74	827.78 ***
		Intention	0.40	[0.36, 0.44]	19.92 ***		
		Outcome	0.52	[0.48, 0.57]	24.91 ***		
Moral Praise (Experiment 1b)	Others-Praise					0.71	665.14 ***
		Intention	0.36	[0.32, 0.40]	17.22 ***		
		Outcome	0.55	[0.51, 0.59]	25.38 ***		
	Self-Praise					0.76	835.16 ***
		Intention	0.36	[0.32, 0.40]	17.71 ***		
		Outcome	0.58	[0.54, 0.62]	27.55 ***		

All the data were standardized. *** *p* < 0.001.

The results of Experiment 2b revealed that regardless of whether evaluations were made based on the first-person or third-person perspective, the behavioural outcome explained significantly greater coefficients of moral praise evaluations than did the behavioural intention factor, supporting Hypothesis 4.

## 4. Study 3: Effects of Intentions and Outcomes on Moral Blame and Praise: Moderating Effects of Self–Other and Outcome Severity

### 4.1. Experiment 3a Effects of Intentions and Outcomes on Moral Blame: Moderating Effects of Self–Other and Outcome Severity

Experiment 3a introduced the variable of outcome severity to Experiment 2a to examine the joint effects of self–other perspectives and outcome severity on the predictions of moral blame from intentions and outcomes.

#### 4.1.1. Participants

In this experiment, the questionnaire was distributed to the participants via the internet. A total of 930 samples were collected, with 467 in the “others” group and 463 in the “self” group (308 males; 622 females; age range: 21.49 ± 3.71 years), and all the participants were compensated for completing the questionnaires and for passing the review of the questionnaires by the main examiner. The recruitment and collection procedures for participants were the same as those in Experiment 1a.

#### 4.1.2. Experimental Design and Procedures

Experiment 3a adopted a 2 (intention: neutral vs. negative) × 2 (outcome severity: minor vs. severe) × 2 (perspective: self vs. other) between-subjects design. Neutral and negative intentions were set up to make intention scores more discriminatory, and intention scores were used as the independent variable in subsequent analyses. A new severe outcome condition was added to the two harmful scenarios from Experiment 2a. Each participant was randomly assigned to one of the eight experimental conditions, each containing both the insecticide and sushi scenarios. After reading each item, the participants completed comprehension, intention, outcome, and severity questions related to the corresponding material and rated the moral blameworthiness of the story protagonist’s behaviour. Specifically, the severity questions indicated the severity of the outcome corresponding to the material on a 7-point scale (1 = not serious, 7 = maximally serious). Additional detailed steps for this questionnaire can be found in Appendix A.

Taking the “sushi” scenario as an example. Neutral intention: Liu Ming believes that the tuna at the sushi restaurant is fresh, and it is safe for colleagues to eat; Negative intention: Liu Ming knows that the tuna at the sushi restaurant is not fresh, and it will be dangerous for colleagues to eat.

Minor outcome: Colleagues have only diarrhoea after eating; Severe outcome: Colleagues suffer from bacterial infection, persistent high fever, and life-threatening conditions after eating.

Others’ perspectives: Evaluate from a third-party perspective: “To what extent should Liu Ming be blamed for his behaviour?”; Self-perspective: “If you were Liu Ming’s colleague, would you blame Liu Ming for his behaviour?”.

#### 4.1.3. Results

Relationships among intention, outcome severity, and blame ratings from the self–other perspective: Hierarchical regression analysis was used to examine the moderating effect of outcome severity on the influence of intention and outcome on moral blame, with standardized blame ratings as the dependent variable. The specific results of the regression model are shown in Table 3.

For others’ perspective judgements: The overall model test revealed that adding interaction terms significantly improved model fit (Δ*R*^2^ = 0.02, Δ*F*(2,461) = 15.10, *p* < 0.001). The Outcome severity × Intention interaction was significant (*p* < 0.001), indicating that outcome severity moderates intention’s effect on blame. Simple slope analysis revealed that intentions more strongly predicted blame under low-severity outcomes compared to high-severity outcomes (*p*s < 0.001). The Outcome severity × Outcome interaction was not significant (*p* = 0.97), indicating that outcome severity did not moderate the effect of outcome on blame.

For self-perspective judgements: Adding interaction terms did not significantly improve model fit (Δ*R*^2^ = 0.004, Δ*F*(2,457) = 1.86, *p* = 0.156). The Outcome Severity × Intention interaction term was not significant (*p* = 0.06). The Outcome Severity × Outcome interaction term was not significant (*p* = 0.85). This indicated that outcome severity did not moderate the predictive effects of intention or outcome on moral blame.

Standardized regressions were performed, with the self–other perspective serving as the grouping variable, intention and outcome ratings serving as independent variables, and moral blame scores serving as the dependent variable.

Intention was a significant positive predictor of moral blame in the other-minor-outcome group ($\beta_{insecticide}$ = 0.46, $\beta_{sushi}$ = 0.61, *p*s < 0.001), and the outcome was also a significant positive predictor of moral blame in this group ($\beta_{insecticide}$ = 0.41, $\beta_{sushi}$ = 0.24, *p*s < 0.001). In the other-severe outcome group, intention was a significant positive predictor of moral blame ($\beta_{insecticide}$ = 0.31, $\beta_{sushi}$ = 0.35, *p*s < 0.001), and the outcome was a significant positive predictor of moral blame ($\beta_{insecticide}$ = 0.38, $\beta_{sushi}$ = 0.36, *p*s < 0.001). In the self-minor outcome group, intention had a significant positive predictive effect on moral blame ($\beta_{insecticide}$ = 0.37, *β*_sushi_ = 0.46, *p*s < 0.001), and the minor outcome had a significant positive predictive effect on moral blame ($\beta_{insecticide}$ = 0.44, $\beta_{sushi}$ = 0.57, *p*s < 0.001). In the self-severe outcome group, intention was a significant positive predictor of moral blame ($\beta_{insecticide}$ = 0.32, $\beta_{sushi}$ = 0.37, *p*s < 0.001), and the severe outcome was a significant positive predictor of moral blame ($\beta_{insecticide}$ = 0.50, $\beta_{sushi}$ = 0.60, *p*s < 0.001).

Standardized regressions were performed with moral blame scores in both contexts as the dependent variable. The positive predictive effect of minor outcomes was significant for both the others’ perspective and the self-perspective (“others” group: *β* = 0.35, 95% CI [0.26, 0.45], *t* = 7.36, *p* < 0.001; “self” group: *β* = 0.54, 95% CI [0.44, 0.65], *t* = 9.94, *p* < 0.001). The positive predictive effect of severe outcomes was also significant (“others” group: *β* = 0.38, 95% CI [0.28, 0.49], *t* = 7.28, *p* < 0.001; “self” group: β = 0.57, 95% CI [0.42, 0.72], *t* = 7.51, *p* < 0.001). Moreover, compared with minor-outcome situations, the blame scores were higher in severe-outcome situations.

By setting α = 0.05, employing sample sizes of 226 (Others-Blame, Minor outcome), 241 (Others-Blame, Severe outcome), 239 (Self-Blame, Minor outcome), and 224 (Self-Blame, Severe outcome),and calculating condition-specific effect sizes, post hoc tests revealed that statistical power reached 1 in both conditions.

From the others’ perspective, the 95% confidence intervals for the coefficients of intention and minor outcomes did not overlap, indicating that intention was a stronger predictor than minor outcomes were. The confidence intervals for the regression coefficients of intention and severe outcome partially overlapped. Thus, there was no significant difference between the regression coefficients of intention and severe outcome, and there was no significant difference between the two predictors of moral blame. However, the beta value indicated a tendency towards the outcome predictor compared with intention. By restricting the model to equate the path coefficients of intention and outcome in moral blame and comparing the two models ($\chi^{2}$ = 822.41, DIF = −0.11, *p* = 0.003), the null hypothesis could be rejected, indicating a significant difference between the two paths of intention and outcome.

From the self-perspective, the 95% confidence intervals for the intention and minor outcome coefficients did not overlap, so the minor outcome was a stronger predictor than the intention. The 95% confidence intervals for the intention and severe outcome coefficients were nonoverlapping; thus, severe outcomes were stronger predictors than were intentions. By restricting the model to equate the path coefficients of intention and outcome in moral blame and comparing the two models ($\chi^{2}$ = 652.21, DIF = −0.23, *p* < 0.001), the null hypothesis could be rejected, revealing a significant difference between the two paths of intention and outcome.

**Table 3:** Linear regression model of intention, minor outcome, and severe outcome on self–other moral blame ratings in the two scenarios.

Outcome Variable	Level of Outcome	Predictors	*β*	95% CI	*t*
1 Others-Blame					
	Minor outcome	Intention	0.53	[0.49, 0.57]	26.05 ***
		Outcome	0.35	[0.26, 0.45]	7.36 ***
	Severe outcome	Intention	0.33	[0.30, 0.37]	18.03 ***
		Outcome	0.38	[0.28, 0.49]	7.28 ***
2 Self-Blame					
	Minor outcome	Intention	0.41	[0.37, 0.46]	18.68 ***
		Outcome	0.54	[0.44, 0.65]	9.94 ***
	Severe outcome	Intention	0.35	[0.30, 0.39]	14.83 ***
		Outcome	0.57	[0.42, 0.72]	7.51 ***

All the data were standardized. *** *p* < 0.001.

The results of Experiment 3a demonstrated that the effects of behavioural intentions and outcomes on moral blame were moderated by the self–other perspective and outcome severity: in moral judgements from the other perspective, people made moral blame evaluations based more on intentions when outcome severity was low, and when outcome severity was high, people made blame evaluations by combining the factors of intentions and outcomes, with no tendency towards self-perspective moral judgement; in self-perspective moral judgement, people made moral blame evaluations prioritize outcomes irrespective of outcome severity under self-perspective, which partially supports Hypothesis 5.

### 4.2. Experiment 3b Effects of Intentions and Outcomes on Moral Praise: The Moderating Role of Self–Other and Outcome Severity

Experiment 3b was based on the story materials from Experiment 2b, with the addition of a severe outcome condition, to examine how self–other perspectives and outcome severity jointly influence the moral praise process.

#### 4.2.1. Participants

In this experiment, the questionnaire was distributed to the participants via the internet. A total of 945 samples were collected, with 464 in the “others” group and 481 in the “self” group (383 males; 562 females; age range: 22.69 ± 4.41 years), and all participants were paid accordingly after completing the questionnaire and passing the review of the questionnaire by the main examiner. The recruitment and collection procedures for participants were the same as those in Experiment 1a.

#### 4.2.2. Experimental Design and Procedures

The experimental design was identical to that of Study 3a. A new severe outcome condition was added to the two helping-scenario materials used in Experiment 2b. Each participant was randomly assigned to one of the eight experimental conditions, each containing two scenarios: the restaurant and the ski resort. The subsequent procedure was the same as that of Experiment 3a. Additional detailed steps for this questionnaire can be found in Appendix A.

Taking the “restaurant” scenario as an example. Neutral intention: Fang Yuan believes that the customer is only choked by chili peppers and is not in danger; Positive intention: Fang Yuan believes that the customer is choking on food and is in danger.

Minor outcome: The customer avoids a wound infection due to Fang Yuan’s reminder; Severe outcome: The customer avoids suffocation and death due to Fang Yuan’s reminder.

Others’ perspectives: Evaluate from a third-party perspective: “To what extent should Fang Yuan’s behaviour be praised?” (judging the degree of praise for Fang Yuan from a third party); Self-perspective: Evaluate “If you were the customer, would you praise Fang Yuan’s behaviour?” (judging whether to praise Fang Yuan when being the customer oneself).

#### 4.2.3. Results

Relationships among intention, outcome severity, and moral praise ratings from the other/self-perspective: Hierarchical regression analysis was used to examine the moderating effect of outcome severity on the influence of intention and outcome on moral praise, with standardized praise ratings as the dependent variable. The specific results of the regression model are shown in Table 4.

For others’ perspective judgements: The overall model test revealed that adding interaction terms did not significantly improved model fit (Δ*R*^2^ = 0.001, Δ*F*(2,458) = 0.01, *p* = 0.97). Both the Outcome Severity × Intention (*p* = 0.92) and Outcome Severity × Outcome (*p* = 0.92) interaction terms were not significant, indicating that outcome severity did not moderate the predictive effects of intention or outcome on moral praise. The main effects of intention and outcome were significant (*p*s < 0.001) and did not change with outcome severity.

For self-perspective judgements: Adding interaction terms significantly improved model fit (Δ*R*^2^ = 0.013, Δ*F*(2,475) = 7.29, *p* < 0.001). The Outcome severity × Intention interaction was significant (*p* < 0.001), indicating that outcome severity moderates intention’s effect on praise. Simple slope analysis revealed that intentions more strongly predicted praise under low-severity outcomes compared to high-severity outcomes (*p*s < 0.001). The Outcome severity × Outcome interaction was not significant (*p* = 0.182), indicating that outcome severity did not moderate the effect of outcome on praise.

Standardized regressions were performed, with the self–other perspective as the grouping variable; intention scores, minor-outcome scores, and severe-outcome scores as independent variables; and moral praise scores as the dependent variable.

Intention was a significant positive predictor of moral praise in the others-minor-outcome group ($\beta_{restaurants}$ = 0.35, $\beta_{ski\;resorts}$ = 0.28, *p*s < 0.001), and outcome was also a significant positive predictor of moral praise in this group ($\beta_{restaurants}$ = 0.24, $\beta_{ski\;resorts}$ = 0.49, *p*s < 0.001). In the others-severe-outcome group, intention was a significant positive predictor of moral praise ($\beta_{restaurants}$ = 0.39, $\beta_{ski\;resorts}$ = 0.25, *p*s < 0.001), and outcome was a significant positive predictor of moral praise ($\beta_{restaurants}$ = 0.16, $\beta_{ski\;resorts}$ = 0.58, *p*s < 0.001). In the self-minor-outcome group, intention was a significant positive predictor of moral praise ($\beta_{restaurants}$ = 0.34, $\beta_{ski\;resorts}$ = 0.16, *p*s < 0.001), and the minor outcome was a significant positive predictor of moral praise ($\beta_{restaurants}$ = 0.35, $\beta_{ski\;resorts}$ = 0.69, *p*s < 0.001). In the self-severe-outcome group, intention was a significant positive predictor of moral praise ($\beta_{restaurants}$ = 0.15, $\beta_{ski\;resorts}$ = 0.10, *p*s < 0.001), and the severe outcome was a significant positive predictor of moral praise ($\beta_{restaurants}$ = 0.57, $\beta_{ski\;resorts}$ = 0.59, *p*s < 0.001).

Standardized regressions were performed with the mean moral praise scores across all three contexts as the dependent variable. In regressions with moral praise as the dependent variable, in both the others’ perspective and the self-perspective, the positive predictive effect of minor outcomes was significant (“others” group: *β* = 0.34, 95% CI [0.26, 0.43], *t* = 7.71, *p* < 0.001; “self” group: *β* = 0.52, 95% CI [0.42, 0.61], *t* = 10.78, *p* < 0.001). The positive predictive effect of severe outcomes was also significant (“others” group: *β* = 0.38, 95% CI [0.28, 0.47], *t* = 8.16, *p* < 0.001; “self” group: *β* = 0.57, 95% CI [0.49, 0.66], *t* = 12.84, *p* < 0.001). Moreover, compared with minor-outcome situations, praise scores were higher in severe-outcome situations.

By setting α = 0.05, employing sample sizes of 232 (Others-Praise, Minor outcome), 232 (Others-Praise, Severe outcome), 240 (Self-Praise, Minor outcome), and 241 (Self- Praise, Severe outcome), and calculating condition-specific effect sizes, post hoc tests revealed that statistical power reached 1 in both conditions.

As shown by the confidence intervals, the confidence intervals for the regression coefficients of intentions and severe outcomes partially overlapped, indicating that there was no significant difference between the predictive effects of intentions and severe outcomes on moral praise. However, the regression coefficients suggested that outcomes played a stronger predictive role than did intentions. Specifically, when the outcome severity was high, the confidence intervals for outcomes were greater than those for intentions, indicating a stronger tendency towards the outcome factor in moral-praise evaluations. By restricting the model to equating the path coefficients of intention and outcome in moral praise and comparing the two models ($\chi^{2}$ = 544.75, DIF = −0.06, *p* = 0.14), there was no significant difference between the two paths of intention and outcome.

From the self-perspective, outcome was a stronger predictor than intention was, regardless of the severity of the outcome. By restricting the model to equate the path coefficients of intention and outcome in moral praise and comparing the two models ($\chi^{2}$ = 550.61, DIF = −0.40, *p* < 0.001), the null hypothesis could be rejected, indicating a significant difference between the two paths of intentions and outcomes.

**Table 4:** Linear regression model of intentions, minor outcomes, and severe outcomes on self–other moral praise ratings in the two scenarios.

Outcome Variable	Level of Outcome	Predictors	*β*	95% CI	*t*
1 Others-Praise					
	Minor outcome	Intention	0.32	[0.27, 0.37]	12.14 ***
		Outcome	0.34	[0.26, 0.43]	7.71 ***
	Severe outcome	Intention	0.31	[0.26, 0.36]	12.68 ***
		Outcome	0.38	[0.28, 0.47]	8.16 ***
2 Self-Praise					
	Minor outcome	Intention	0.25	[0.20, 0.29]	10.54 ***
		Outcome	0.52	[0.42, 0.61]	10.78 ***
	Severe outcome	Intention	0.13	[0.09, 0.17]	6.30 ***
		Outcome	0.57	[0.49, 0.66]	12.84 ***

All the data were standardized. *** *p* < 0.001.

The results of Experiment 3b showed that in moral judgement from others’ perspectives, regardless of the severity of the outcome, people made praise evaluations on the basis of a combination of intent and outcome factors without a tendency. In moral judgements from the self-perspective, regardless of the severity of the outcome, people made praise evaluations based more on the outcome.

## 5. Discussion

Cushman (2008) proposed a dual-process theory of moral judgement based on moral blame and punishment judgements, namely, intent-based and outcome-based processes. Although prior research has documented numerous asymmetries between moral blame and praise (Bostyn & Roets, 2016; Cushman & Greene, 2012; Pizarro et al., 2003), the issue of whether and how intentions and outcomes have asymmetric effects on blame and praise remains underexplored. The present study investigated the effects of intention-outcome on blame and praise from an intention-outcome two-dimensional perspective, using a classic intention-outcome judgement task adapted from previous story material and investigated how the process is moderated by self–other perspectives and outcome severity. This study explained the relative contributions of intention and outcome factors in the evaluation of blame and praise by analysing the differences in the weights of the predictive coefficients of intention and outcome in a linear regression model (Cumming, 2009), and the results indicated that there was an asymmetric effect of behavioural intention-outcome on moral blame and praise. Intention was more highly valued in the process of moral blame, whereas outcomes were more highly valued in the process of moral praise. When the judgement perspective shifted from the other’s perspective to the self-perspective, both blame and praise placed more emphasis on outcomes. When the outcome severity level changed from minor to severe, intention became a weaker predictor of blame and praise (however, this effect was only observed in moral blame from others’ perspective, and moral praise from the self-perspective), while the predictive role of outcome for blame and praise remained unaffected.

### 5.1. Asymmetric Effects of Behavioural Intentions-Outcomes on Moral Blame and Praise

The social-functional model explains asymmetries in how intentions and outcomes influence moral blame and praise (Anderson et al., 2020; Carnes et al., 2022; Malle, 2006). Social functionalism states that moral judgement plays a critical role in maintaining and regulating social relations and cooperation among society members. In this context, moral blame and praise play related yet distinct roles in social regulation (Curry, 2016; Haidt, 2008; Rai & Fiske, 2011). The primary function of blame is to regulate behaviour through criticism and is thus subject to social norms. Therefore, the blamer must provide compelling reasons to justify why the actor deserves blame (Coates & Tognazzini, 2012; McKenna, 2012). Compared with blame, praise plays a greater role in building and maintaining social relationships (Algoe et al., 2016; Schein et al., 2020). Moreover, when incorrect praise and blame judgements are made, different costs are incurred. For the blamer, making wrongful accusations may result in varying degrees of social ostracism, damage or rupture of their social relationships, and even physical punishment and retaliation by others (Dreber et al., 2008; McCullough et al., 2013). Thus, blame regarded as incorrect or misjudged can lead to resentment as well as psychological harm due to the injustice experienced by the recipient of blame (Schein et al., 2020; Aquino et al., 2001). In contrast to blame, false praise seems to be cost-free for both the praiser and the recipient. Thus, praise is relatively insensitive to the fine-grained analytic processes of intent, causality, and control in blame judgements (Pizarro et al., 2003).

From the perspective of processing mechanisms, the processing of helping behaviour, as a positive event, may differ from that of negative events. This difference may stem from the fact that people have different processing mechanisms for positive and negative stimuli (Baumeister et al., 2001). For example, the negative bias effect has been identified in the field of emotion research, where studies have shown that people are more sensitive to negative emotional events (Huang & Luo, 2006). In the field of morality research, Pizarro et al. (2003) reported significant differences in the criteria and process mechanisms by which individuals evaluate moral events positively and negatively. The Knobe effect indicates that information about the perpetrator’s negative intentions guides moral judgements more significantly than information about the giver’s positive intentions does (Knobe, 2003; Nobes et al., 2009). From a social norm perspective, positive intentions are considered social norms, whereas negative intentions are viewed as deviations from these norms, as individuals generally default to assuming that actors have positive behavioural intentions in social contexts (Levine et al., 2018).

### 5.2. Asymmetric Effects of Behavioural Intentions and Outcomes on Moral Blame and Praise: Self–Other Perspective Differences

Previous researchers have explained the self–other decision-making perspective from emotional and dual-system perspectives (Beisswanger et al., 2003; Sun et al., 2016), proposing that individuals are more emotionally engaged when making decisions for themselves than when making decisions for others, leading to variability in decision-making. Albrecht et al. (2011) reported that when individuals make decisions for themselves compared with making decisions for others, the brain regions responsible for emotions show higher levels of activation. Moreover, at a physiological level, it was found that intention-based moral judgements are more demanding of cognitive resources than outcome-based moral judgements are (Young et al., 2010; Buon et al., 2013; Martin et al., 2022). Indeed, intent-based moral judgement processes do not rely purely on cognitive processing systems, and both outcome- and intent-based processes involve emotional content of different psychological origins (Hauser, 2006; Huebner et al., 2009). On the one hand, outcome-based blame processes generate emotional aversion derived from negative outcomes, termed “outcome aversion,” which involves mentally simulating the pain suffered by the victim (Hoffman, 2001; Pizarro, 2000). On the other hand, intent-based reasoning produces “behavioural aversions” derived from negative intentions, regardless of outcomes (Blair, 2007; Cushman et al., 2013; Crockett, 2013). These aversions may stem from abstract categorizations of negative behaviours (e.g., “Actor A intends to harm B”) (Miller et al., 2010) without further reflection on whether these behaviours ultimately lead to harm. From this perspective, outcome-based emotional aversion in the blame process may be more prominent from the self-perspective, whereas behaviour-based emotional aversion may be more prominent from the others’ perspective. This thus leads to moral blame from the self-perspective prioritizing outcomes, and moral blame from the other perspective prioritizing intentions.

The finding of this study, that blame from the others’ perspective prioritizes intent while blame from the self-perspective prioritizes outcomes, is consistent with the determination of liability in legal practice. Individuals often evade responsibility for inappropriate behaviours by claiming “I didn’t mean to do it” or similar justifications. However, Schleim et al. (2011) found that in legal practice, third parties such as judges strictly rely on intention inferences when distinguishing between “intentional” and “negligent” acts, whereas parties involved tend to downplay intentions to evade responsibility. Specifically, there is a discrepancy in attribution between parties involved and third parties. For parties involved, emphasizing “non-intentionality” is a self-protective strategy; they alleviate self-blame by attributing responsibility to uncontrollable outcomes (Cushman, 2008). For third parties, however, intent is the core basis for judging responsibility. In legal practice, the distinction between “intentional” and “negligent” directly affects sentencing; therefore, third parties are more inclined to infer intentions backward from behavioural outcomes (Schleim et al., 2011).

Furthermore, the positive–negative mood mobilization asymmetry indicates that those in positive moods induced by positive events use more heuristic judgements to solve problems (Clark & Isen, 1982), make decisions faster and use less information (Isen & Means, 1983) than those in neutral moods do. In contrast, negative emotions produce more collection of diagnostic information (Hildebrand-Saints & Weary, 1989), more chunking of information (Isen et al., 1987; Leight & Ellis, 1981), more complex processing strategies, less use of cognitive heuristics, and more systematic elaboration of complex information (Bless et al., 1990; Fiedler, 1988; Clark & Isen, 1982; Schwarz, 1990; Sinclair, 1988). In the process of praise, either positive outcomes or positive intentions can generate positive emotions, which play a more prominent role from the self-perspective, so that a clear preference for outcome factors emerges when participants make moral praise evaluations, when they may have used heuristic information processing. According to the phenomenon of positive–negative emotion mobilization asymmetry, moral blame and praise are affected by the asymmetry of positive–negative emotions from the self’s and others’ perspectives, which may have led to the adoption of different processing strategies, and future research is needed to clarify whether there is a difference in this processing mechanism.

### 5.3. Asymmetric Effects of Behavioural Intentions and Outcomes on Moral Blame and Praise: The Moderating Role of Outcome Severity

Findings in moral psychology suggested that individuals tend to make judgements on the basis of more salient negative information in a situation due to the influence of negative bias in making moral judgements (Cowan & Yazdanparast, 2019), which makes outcome severity an important consideration when making moral judgement assessments, i.e., as the severity of the harm caused by the perpetrator to the victim increased, people were more inclined to punish the individual on the basis of the outcome (Carlsmith et al., 2002; Robbennolt, 2000).

Study 3 introduced the outcome-severity variable to investigate how self–other perspectives and outcome severity moderated the asymmetric effects of intentions and outcomes on moral blame and praise. The study found that outcome severity moderated the effect of behavioural intentions on moral blame and moral praise but did not moderate the effect of outcomes on moral blame, which partially supported Hypothesis 5. When outcomes were severe, it did not directly enhance the importance of outcomes for moral judgements; instead, it indirectly enhanced the predictive power of outcomes for moral judgements by reducing the predictive power of intentions for moral blame and moral praise. However, this effect was only observed in moral blame from the others’ perspective and moral praise from the self-perspective.

Research has revealed that there may be a “harm severity threshold” in an individual’s psyche and that when the severity exceeds this threshold, the emotional load generated by severe harm outcomes may limit intention-based analysis (Schwartz et al., 2022), which may account for why outcome severity reduces the predictive power of intentions for moral blame and moral praise. Research indicates that victims tend to exaggerate the injustice they have suffered and perceive the consequences as more severe (Baumeister, 1999). Due to victims’ attention being affected by the focalization effect (Adams & Inesi, 2016; McCullough et al., 2003), they are more prone to being influenced by outcome severity than bystanders. Consequently, the moderating effect of outcome severity on the importance of intent was only observed in moral blame scenarios from others’ perspectives in this study.

The results of Study 3 regarding praise from the perspective of others were not entirely consistent with the those of Study 2. There was no intention or outcome bias for praise at either mild or severe levels of outcome from the other perspective. The results of Study 2, on the other hand, indicated that praise from others’ perspectives was more outcome-oriented. However, the direction and trend of the data revealed that the two studies were consistent. Therefore, differences in study design and limitations in data analysis methods may have contributed to the inconsistency of the results, and further validation is needed in the future.

### 5.4. Significance of the Study

This study revealed the asymmetric effects of intentions and outcomes on moral blame and praise using a classic intention-outcome judgement task adapted to helper-related contexts. It also examined the asymmetric processes of moral blame and praise from a self–other perspective, contributing to the literature on self–other decision-making differences in ethics. Moreover, given that everyday moral judgements involve diverse types of harm beyond death (e.g., physical harm, emotional harm, financial loss) (Gold, 2013), this research introduced the outcome–severity variable to investigate, for the first time, how self–other perspectives and outcome severity moderate the asymmetric effects of intention–outcome on moral blame and praise. These findings suggest that in conflict mediation or legal adjudication, third parties need to be wary of the self-serving bias of involved parties and to more objectively evaluate the weights of intentions and outcomes. For example, judges should integrate behavioural outcomes and subjective intentions during sentencing, avoiding over-reliance on information from a single dimension.

With the development of internet technology, people are increasingly exposed to moral events through news media. In this context, emphasizing and deepening research on moral evaluation is particularly important. This study helps individuals understand their own tendencies when evaluating behaviour or judging others, thereby facilitating more objective and rational assessments. At the social level, by publicizing the visible positive significance of good deeds, helpers’ self-inhibition can be alleviated, and the dissemination of altruistic behaviours can be promoted. Additionally, this study underscores the importance of recognizing that even seemingly trivial good deeds should not be overlooked.

### 5.5. Research Limitations and Future Research Perspectives

This study investigated the asymmetric effects of intentions and outcomes on moral blame and praise using an intention–outcome judgement task. In this paradigm, story materials are presented in the sequence “background-foreshadowing-intention-behaviour and outcome,” potentially leading participants to precode intentional information before fully processing the behavioural events. Therefore, future research could manipulate the order of information presentation and visual salience to better clarify the relative contributions of intention and outcome. In addition, based on Cushman’s (2008) findings of intention prioritization in blame and outcome prioritization in punishment, future studies should further examine whether similar asymmetries exist in the praise and reward processes. Moral foundations theory (Haidt & Joseph, 2004) identifies five moral foundations: harm/care, fairness/reciprocity, in-group/loyalty, authority/respect, and purity/sanctity. Given the distinct cognitive profiles of intentions in different moral domains within individuals and societies, future research should use multiple types of moral situations (e.g., unfairness, unkindness, honesty, jealousy, forgiveness) and response types (e.g., resentment, punishment, reward) to further explore the dynamics of intent-consequence processes influencing moral judgements and the underlying reasons.

Finally, all data in this study were collected via self-report measures, which presents certain limitations. Self-reports are susceptible to social desirability bias, wherein participants may provide responses aligned with moral norms rather than their genuine judgements. Moreover, quantitative ratings capture only the final moral evaluation and not the underlying cognitive processes (e.g., hesitation or weighting of intention versus outcome). Additionally, the sample was recruited online and consisted primarily of younger adults, limiting the generalizability of the findings to other age groups such as children and older adults. Future research could employ process-tracing techniques (e.g., mouse tracking; Gaboriaud et al., 2022) to dynamically capture moral decision-making processes and further examine the asymmetries identified in this study.

## 6. Conclusions

We investigated the asymmetric effects of behavioural intentions and outcomes on moral blame and praise, moderated by self–other perspectives and outcome severity, through three experiments.

The overall results demonstrated that moral blame prioritized behavioural intentions, while moral praise emphasized behavioural outcomes. The self–other perspective further moderated this pattern: from others’ perspectives, intention had a stronger predictive effect on blame. From the self-perspective, outcome had a stronger predictive effect on blame. For praise, outcome served as a more important basis for judgement in both perspectives; outcome severity moderated the effect of behavioural intentions on both moral blame and moral praise but did not moderate the effect of outcome on moral blame; specifically, intention had a stronger predictive effect on blame and praise when outcomes were minor compared to when they were severe. This study reveals the dynamic interactions among factors such as intention, outcome, perspective, and outcome severity in moral evaluation, providing a research foundation for an in-depth understanding of the asymmetry between moral blame and praise.

## Data Availability

The original contributions presented in this study are included in the article. Further inquiries can be directed to the corresponding author.

## Appendix A

### Appendix A.1. “Others” Group on Moral Blame

Taking the “insecticide” scenario in Experiment 3a as an example.

**Neutral Intention—Minor Outcome**

On Saturday afternoon, Sun Yao carried out pest control work at home and sprayed a lot of insecticide in the house.

Sun Yao’s neighbour was sleeping in the upstairs room and wasn’t woken up even though Sun Yao was spraying the insecticide.

The neighbour had told Sun Yao that she would go out early on Saturday, so Sun Yao thought the neighbour wasn’t in the room and that spraying the insecticide wouldn’t harm the neighbour.

Sun Yao sprayed the whole house with insecticide, and her neighbor felt a headache after inhaling too much of it.

1. **Comprehension Questions (Yes/No)**

Did Sun Yao think that her neighbour would be harmed by the insecticide? (No)

Was Sun Yao’s neighbour harmed by the insecticide? (Yes)

2. **Intentionality Question (1–7)**

To what extent did Sun Yao intend to harm her neighbour with the insecticide?

3. **Outcome-related Question (1–7)**

To what extent was Sun Yao’s neighbour harmed?

4. **Moral Blame Evaluation (1–7)**

To what extent should Sun Yao’s behaviour be blamed?

**Neutral Intention—Severe Outcome**

On Saturday afternoon, Sun Yao carried out pest control work at home and sprayed a lot of insecticide in the house.

Sun Yao’s neighbour was sleeping in the upstairs room and wasn’t woken up even though Sun Yao was spraying the insecticide.

The neighbour had told Sun Yao that she would go out early on Saturday, so Sun Yao thought the neighbour wasn’t in the room and that spraying the insecticide wouldn’t harm the neighbour.

Sun Yao sprayed the whole house with insecticide, and her neighbour suffered from acute poisoning and went into shock after inhaling too much of it.

(Specific questions as above.)

**Negative Intention—Minor Outcome**

On Saturday afternoon, Sun Yao carried out pest control work at home and sprayed a lot of insecticide in the house.

Sun Yao’s neighbour was sleeping in the upstairs room and wasn’t woken up even though Sun Yao was spraying the insecticide.

Sun Yao’s neighbour seldom goes out on weekends, so Sun Yao thought the neighbour was in the room and that spraying the insecticide would harm the neighbour.

Sun Yao sprayed the whole house with insecticide, and her neighbour felt a headache after inhaling too much of it.

(Specific questions as above.)

**Negative Intention-Severe Outcome**

On Saturday afternoon, Sun Yao carried out pest control work at home and sprayed a lot of insecticide in the house.

Sun Yao’s neighbour was sleeping in the upstairs room and wasn’t woken up even though Sun Yao was spraying the insecticide.

Sun Yao’s neighbour seldom goes out on weekends, so Sun Yao thought the neighbour was in the room and that spraying the insecticide would harm the neighbour.

Sun Yao sprayed the whole house with insecticide, and her neighbour suffered from acute poisoning and went into shock after inhaling too much of it.

(Specific questions as above.)

### Appendix A.2. “Self” Group on Moral Praise

Taking the “Restaurant” scenario in Experiment 3b as an example

**Neutral Intention-Minor Outcome**

Sun Yao is your neighbour. On Saturday afternoon, Sun Yao carried out pest control work at home and sprayed a lot of insecticide in the house.

One noon, Fang Yuan made an appointment to have lunch with a doctor friend. Fang Yuan arrived at the restaurant first. At this time, you suddenly let out a scream.

Your hand, which had just been redressed, was accidentally scalded by the tea. It needed to be redressed immediately, otherwise, there was a risk of wound infection.

Fang Yuan didn’t notice that your hand had been injured before, so Fang Yuan thought you were not in danger.

After the doctor friend arrived, Fang Yuan told the doctor friend about your situation. The doctor friend realized something was wrong and redressed your wound, so you didn’t get infected.

1. **Comprehension Questions (Yes/No)**

Did Fang Yuan think you were in danger? (No)

Were you in danger? (Yes)

2. **Intentionality Question (1–7)**

To what extent did Fang Yuan intend to help you get out of danger?

3. **Outcome-related Question (1–7)**

To what extent were you helped?

4. **Moral Praise Evaluation (1–7)**

Would you praise Fang Yuan’s behaviour?

**Neutral Intention-Severe Outcome**

One noon, Fang Yuan made an appointment to have lunch with a doctor friend. Fang Yuan arrived at the restaurant first. At this time, you suddenly started coughing.

You accidentally had a piece of beef stuck in your trachea and needed immediate first-aid. There was a risk of suffocation.

Because this was a Sichuan-style restaurant, Fang Yuan thought you just ate something very spicy, and the situation was safe.

After the doctor friend arrived, Fang Yuan casually told the doctor friend about your situation. The doctor friend realized something was wrong and gave you first-aid, so you were saved.

(Specific questions as above.)

**Positive Intention—Minor Outcome**

One noon, Fang Yuan made an appointment to have lunch with a doctor friend. Fang Yuan arrived at the restaurant first. At this time, you suddenly let out a scream.

Your hand, which had just been redressed, was accidentally scalded by the tea. It needed to be redressed immediately, otherwise, there was a risk of wound infection.

Fang Yuan noticed that your hand had been injured before, so Fang Yuan thought you were in danger.

After the doctor friend arrived, Fang Yuan immediately asked the doctor friend to redress your wound, so you didn’t get infected.

(Specific questions as above.)

**Positive Intention—Severe Outcome**

One noon, Fang Yuan made an appointment to have lunch with a doctor friend. Fang Yuan arrived at the restaurant first. At this time, you suddenly started coughing.

You accidentally had a piece of beef stuck in your trachea and needed immediate first-aid. There was a risk of suffocation.

Fang Yuan thought you had your trachea blocked by food and the situation was dangerous.

After the doctor friend arrived, Fang Yuan immediately asked the doctor friend to give you first-aid, so you were saved.

(Specific questions as above.)

## References

[B1-behavsci-15-01265] Adams G. S., Inesi M. E. (2016). Impediments to forgiveness: Victim and transgressor attributions of intent and guilt. Journal of Personality and Social Psychology.

[B2-behavsci-15-01265] Agerström J., Björklund F., Carlsson R. (2013). Look at yourself! Visual perspective influences moral judgment by level of mental construal. Social Psychology.

[B3-behavsci-15-01265] Albrecht K., Volz K. G., Sutter M., Laibson D. I., von Cramon D. Y. (2011). What is for me is not for you: Brain correlates of intertemporal choice for self and others. Social Cognitive and Affective Neuroscience.

[B4-behavsci-15-01265] Algoe S. B., Kurtz L. E., Hilaire N. M. (2016). Putting the “You” in “Thank You”: Examining other-praising behavior as the active relational ingredient in expressed gratitude. Social Psychological and Personality Science.

[B5-behavsci-15-01265] Alicke M. D. (2000). Culpable control and the psychology of blame. Psychological Bulletin.

[B6-behavsci-15-01265] Anderson R. A., Crockett M. J., Pizarro D. A. (2020). A theory of moral praise. Trends in Cognitive Sciences.

[B7-behavsci-15-01265] Aquino K., Tripp T. M., Bies R. J. (2001). How employees respond to personal offense: The effects of blame attribution, victim status, and offender status on revenge and reconciliation in the workplace. Journal of Applied Psychology.

[B8-behavsci-15-01265] Baumeister R. F. (1999). The self in social psychology.

[B9-behavsci-15-01265] Baumeister R. F., Bratslavsky E., Finkenauer C., Vohs K. D. (2001). Bad is stronger than good. Review of General Psychology.

[B10-behavsci-15-01265] Beisswanger A. H., Stone E. R., Hupp J. M., Allgaier L. (2003). Risk taking in relationships: Differences in deciding for oneself versus for a friend. Basic and Applied Social Psychology.

[B11-behavsci-15-01265] Blair R. J. R. (2007). The amygdala and ventromedial prefrontal cortex in morality and psychopathy. Trends in Cognitive Sciences.

[B12-behavsci-15-01265] Bless H., Bohner G., Schwarz N., Strack F. (1990). Mood and persuasion: A cognitive response analysis. Personality and Social Psychology Bulletin.

[B13-behavsci-15-01265] Bostyn D. H., Roets A. (2016). The morality of action: The asymmetry between judgments of praise and blame in the action-omission effect. Journal of Experimental Social Psychology.

[B14-behavsci-15-01265] Buon M., Jacob P., Loissel E., Dupoux E. (2013). A non-mentalistic cause-based heuristic in human social evaluations. Cognition.

[B15-behavsci-15-01265] Carlsmith K. M., Darley J. M., Robinson P. H. (2002). Why do we punish? Deterrence and just deserts as motives for punishment. Journal of Personality and Social Psychology.

[B16-behavsci-15-01265] Carnes N. C., Allmon B., Alva J., Cousar K. A., Varnam Z. D. (2022). How morality signals, benefits, binds and teaches. Journal of Experimental Social Psychology.

[B17-behavsci-15-01265] Clark M. S., Isen A. M. (1982). Toward understanding the relationship between feeling states and social behavior. Cognitive Social Psychology.

[B19-behavsci-15-01265] Coates D. J., Tognazzini N. A. (2012). The nature and ethics of blame. Philosophy Compass.

[B18-behavsci-15-01265] Costa-Lopes R., Mata A., Mendonça C. (2021). Real people or mere numbers? The influence of kill-save ratios and identifiability on moral judgments. International Journal of Social Psychology.

[B20-behavsci-15-01265] Cowan K., Yazdanparast A. (2019). Moral foundations and judgment: Conceptualizing boundaries. Journal of Consumer Marketing.

[B21-behavsci-15-01265] Crockett M. J. (2013). Models of morality. Trends in Cognitive Sciences.

[B23-behavsci-15-01265] Cumming G. (2009). Inference by eye: Reading the overlap of independent confidence intervals. Statistics in Medicine.

[B22-behavsci-15-01265] Curry O. S. (2016). Morality as cooperation: A problem-centred approach. The evolution of morality.

[B24-behavsci-15-01265] Cushman F. (2008). Crime and punishment: Distinguishing the roles of causal and intentional analyses in moral judgment. Cognition.

[B25-behavsci-15-01265] Cushman F. (2015). Deconstructing intent to reconstruct morality. Current Opinion in Psychology.

[B26-behavsci-15-01265] Cushman F., Greene J. D. (2012). Finding faults: How moral dilemmas illuminate cognitive structure. Social Neuroscience.

[B27-behavsci-15-01265] Cushman F., Sheketoff R., Wharton S., Carey S. (2013). The development of intent-based moral judgment. Cognition.

[B28-behavsci-15-01265] Dreber A., Rand D. G., Fudenberg D., Nowak M. A. (2008). Winners don’t punish. Nature.

[B29-behavsci-15-01265] Duan L., Mo S., Fan C., Liu H. (2012). The role of mental states and event causation in moral judgment: A test of the dual-processing theory of moral judgment. Psychological Journal.

[B30-behavsci-15-01265] Fiedler K. (1988). The dependence of the conjunction fallacy on subtle linguistic factors. Psychological Research.

[B31-behavsci-15-01265] Gaboriaud A., Gautheron F., Quinton J.-C., Smeding A. (2022). The effects of Intent, outcome, and causality on moral judgments and decision processes. Psychologica Belgica.

[B32-behavsci-15-01265] Gan T., Lu X., Li W., Gui D., Tang H., Mai X., Liu C., Luo Y.-J. (2016). Temporal dynamics of the integration of intention and outcome in harmful and helpful moral judgment. Frontiers in Psychology.

[B33-behavsci-15-01265] Gan T., Shi R., Liu C., Luo Y. (2018). Effects of transcranial direct current stimulation of the right temporoparietal joint area on the processing of helping intention. Psychological Journal.

[B34-behavsci-15-01265] Gold N., Pulford B. D., Colman A. M. (2013). Your money or your life: Comparing judgements in trolley problems involving economic and emotional harms, injury and death. Economics & Philosophy.

[B35-behavsci-15-01265] Green D. M., Swets J. A. (1996). Signal detection and psychophysics.

[B36-behavsci-15-01265] Greene J., Haidt J. (2002). How (and where) does moral judgment work?. Trends in Cognitive Sciences.

[B38-behavsci-15-01265] Haidt J. (2008). Morality. Perspectives on Psychological Science.

[B39-behavsci-15-01265] Haidt J., Joseph C. (2004). Intuitive ethics: How innately prepared intuitions generate culturally variable virtues. Daedalus.

[B40-behavsci-15-01265] Hauser M. (2006). Moral minds: How nature designed our universal sense of right and wrong.

[B41-behavsci-15-01265] Hildebrand-Saints L., Weary G. (1989). Depression and social information gathering. Personality and Social Psychology Bulletin.

[B43-behavsci-15-01265] Hoffman M. L., Bohart A. C., Stipek D. J. (2001). Toward a comprehensive empathy-based theory of prosocial moral development. Constructive & destructive behavior: Implications for family, school, & society.

[B44-behavsci-15-01265] Huang Y. X., Luo Y. J. (2006). Temporal course of emotional negativity bias: An ERP study. Neuroscience Letters.

[B45-behavsci-15-01265] Huebner B., Dwyer S., Hauser M. (2009). The role of emotion in moral psychology. Trends in cognitive sciences.

[B47-behavsci-15-01265] Isen A. M., Daubman K. A., Nowicki G. P. (1987). Positive affect facilitates creative problem solving. Journal of Personality and Social Psychology.

[B46-behavsci-15-01265] Isen A. M., Means B. (1983). The influence of positive affect on decision-making strategy. Social Cognition.

[B37-behavsci-15-01265] Kahneman D., Tversky A. (1984). Choices, values, and frames. American Psychologist.

[B42-behavsci-15-01265] Knobe J. (2003). Intentional action and side effects in ordinary language. Analysis.

[B48-behavsci-15-01265] Kurdi B., Krosch A. R., Ferguson M. J. (2020). Implicit evaluations of moral agents reflect intent and outcome. Journal of Experimental Social Psychology.

[B49-behavsci-15-01265] Lammers J. (2012). Abstraction increases hypocrisy. Journal of Experimental Social Psychology.

[B50-behavsci-15-01265] Leight K. A., Ellis H. C. (1981). Emotional mood states, strategies, and state-dependency in memory. Journal of Verbal Learning and Verbal Behavior.

[B51-behavsci-15-01265] Levine E. E., Bitterly T. B., Cohen T. R., Schweitzer M. E. (2018). Who is trustworthy? Predicting trustworthy intentions and behavior. Journal of Personality and Social Psychology.

[B52-behavsci-15-01265] Liu Y. F., Bi Y. F., Wang H. Y. (2010). The effects of emotion and task framing on risk preferences in self- and anticipatory-other decision making. Psychological Journal.

[B53-behavsci-15-01265] Lowe C. A., Medway F. J. (1976). Effects of valence, severity, and relevance on responsibility and dispositional attribution. Journal of Personality.

[B54-behavsci-15-01265] Malle B. F. (2006). Intentionality, morality, and their relationship in human judgment. Journal of Cognition and Culture.

[B55-behavsci-15-01265] Malle B. F., Guglielmo S., Monroe A. E. (2014). A theory of blame. Psychological Inquiry.

[B56-behavsci-15-01265] Malle B. F., Guglielmo S., Voiklis J., Monroe A. E. (2022). Cognitive blame is socially shaped. Current Directions in Psychological Science.

[B57-behavsci-15-01265] Malle B. F., Knobe J. (1997). The folk concept of intentionality. Journal of Experimental Social Psychology.

[B59-behavsci-15-01265] Martin J. W., Buon M., Cushman F. (2021). The effect of cognitive load on intent-based moral judgment. Cognitive Science.

[B58-behavsci-15-01265] Martin J. W., Cushman F. (2016). Why we forgive what can’t be controlled. Cognition.

[B60-behavsci-15-01265] Mata A. (2019). Social metacognition in moral judgment: Decisional conflict promotes perspective taking. Journal of Personality and Social Psychology.

[B62-behavsci-15-01265] McCullough M. E., Fincham F. D., Tsang J. A. (2003). Forgiveness, forbearance, and time: The temporal unfolding of transgression-related interpersonal motivations. Journal of Personality and Social Psychology.

[B61-behavsci-15-01265] McCullough M. E., Kurzban R., Tabak B. A. (2013). Cognitive systems for revenge and forgiveness. Behavioral and Brain Sciences.

[B63-behavsci-15-01265] McKenna M. (2012). Conversation & responsibility.

[B64-behavsci-15-01265] Miller S. L., Maner J. K., Becker D. V. (2010). Self-protective biases in group categorization: Threat cues shape the psychological boundary between “us” and “them”. Journal of Personality and Social Psychology.

[B65-behavsci-15-01265] Moran J. M., Young L. L., Saxe R., Lee S. M., O’Young D., Mavros P. L., Gabrieli J. D. (2011). Impaired theory of mind for moral judgment in high-functioning autism. Proceedings of the National Academy of Sciences.

[B66-behavsci-15-01265] Nobes G., Panagiotaki G., Pawson C. (2009). The influence of negligence, intention, and outcome on children’s moral judgments. Journal of Experimental Child Psychology.

[B67-behavsci-15-01265] Ohtsubo Y. (2007). Perceived intentionality intensifies blameworthiness of negative behaviors: Blame-praise asymmetry in intensification effect. Japanese Psychological Research.

[B68-behavsci-15-01265] Peeters G., Czapinsky J. (1990). Positive-negative asymmetry in evaluations: The distinction between affective and informational negativity effects. European Review of Social Psychology.

[B69-behavsci-15-01265] Pizarro D. (2000). Nothing more than feelings? The role of emotions in moral judgment. Journal for the Theory Of Social Behaviour.

[B70-behavsci-15-01265] Pizarro D., Uhlmann E., Salovey P. (2003). Asymmetry in judgments of moral blame and praise: The role of perceived mental desires. Psychological Science.

[B71-behavsci-15-01265] Polman E. (2012). Effects of self-other decision making on regulatory focus and choice overload. Journal of Personality and Social Psychology.

[B72-behavsci-15-01265] Rai T. S., Fiske A. P. (2011). Moral psychology is relationship regulation: Moral motives for unity, hierarchy, equality, and proportionality. Psychological Review.

[B73-behavsci-15-01265] Robbennolt J. K. (2000). Outcome severity and judgments of “responsibility”: A meta-analytic review 1. Journal of Applied Social Psychology.

[B74-behavsci-15-01265] Rozin P., Royzman E. B. (2001). Negativity bias, negativity dominance, and contagion. Personality and Social Psychology Review.

[B75-behavsci-15-01265] Schein C., Gray K. (2018). The theory of dyadic morality: Reinventing moral judgment by redefining harm. Personality and Social Psychology Review.

[B76-behavsci-15-01265] Schein C., Jackson J. C., Frasca T., Gray K. (2020). Praise-many, blame-fewer: A common (and successful) strategy for attributing responsibility in groups. Journal of Experimental Psychology: General.

[B77-behavsci-15-01265] Schleim S., Spranger T. M., Erk S., Walter H. (2011). From moral to legal judgment: The influence of normative context in lawyers and other academics. Social Cognitive and Affective Neuroscience.

[B79-behavsci-15-01265] Schwartz F., Djeriouat H., Trémolière B. (2022). Judging accidental harm: Reasoning style modulates the weight of intention and harm severity. Quarterly Journal of Experimental Psychology.

[B78-behavsci-15-01265] Schwarz N. (1990). Feelings as information: Informational and motivational functions of affective states.

[B80-behavsci-15-01265] Sinclair R. C. (1988). Mood, categorization breadth, and performance appraisal: The effects of order of information acquisition and affective state on halo, accuracy, information retrieval, and evaluations. Organizational Behavior and Human Decision Processes.

[B81-behavsci-15-01265] Song H. R., Chen L. C., Zhao R. K. (1989). Ethics dictionary.

[B82-behavsci-15-01265] Sun H. Y., Cui L. Y., Li D. (2016). Negative discounting phenomenon: Self-other decision-making differences in intertemporal decision-making. Psychological Science.

[B83-behavsci-15-01265] Trope Y., Liberman N. (2000). Temporal construal and time-dependent changes in preference. Journal of Personality and Social Psychology.

[B84-behavsci-15-01265] Wang T., Jin S., Cheng Z., Lou Y., Xie X. (2024). Prediction bias in conspicuous altruism: Helpers underestimate social evaluations from bystanders. Acta Psychologica Sinica.

[B85-behavsci-15-01265] Young L., Bechara A., Tranel D., Damasio H., Hauser M., Damasio A. (2010). Damage to ventromedial prefrontal cortex impairs judgment of harmful intent. Neuron.

[B86-behavsci-15-01265] Young L., Cushman F., Hauser M., Saxe R. (2007). The neural basis of the interaction between theory of mind and moral judgment. Proceedings of the National Academy of Sciences.

[B87-behavsci-15-01265] Young L., Scholz J., Saxe R. (2011). Neural evidence for “intuitive prosecution”: The use of mental state information for negative moral verdicts. Social Neuroscience.

